# Profiling intestinal stem and proliferative cells in the small intestine of broiler chickens via in situ hybridization during the peri-hatch period

**DOI:** 10.1016/j.psj.2023.102495

**Published:** 2023-01-13

**Authors:** Sara E. Cloft, Zehava Uni, Eric A. Wong

**Affiliations:** ⁎ School of Animal Sciences, Virginia Tech, Blacksburg, VA 24061, USA; † Department of Animal Science, The Robert H. Smith, Faculty of Agriculture, Food and Environment, The Hebrew University of Jerusalem, Rehovot 76100, Israel

**Keywords:** small intestine, stem cell, Lgr5, Olfm4, Ki67

## Abstract

Mature small intestines have crypts populated by stem cells which produce replacement cells to maintain the absorptive villus surface area. The embryonic crypt is rudimentary and cells along the villi are capable of proliferation. By 7 d post-hatch the crypts are developed and are the primary sites of proliferation. Research characterizing the proliferative expansion of the small intestine during the peri-hatch period is lacking. The objective of this study was to profile the changes of genes that are markers of stem cells and proliferation: Olfactomedin 4 (**Olfm4**), Leucine-rich repeat containing G protein-coupled receptor 5 (**Lgr5**), and marker of proliferation Ki67 from embryonic day 17 to 7 d post-hatch using quantitative PCR and in situ hybridization (**ISH**). The expression of the stem cell marker genes differed. Olfm4 mRNA increased while Lgr5 mRNA decreased post-hatch. Ki67 mRNA decreased post-hatch in the duodenum and was generally the greatest in the ileum. The ISH was consistent with the quantitative PCR results. Olfm4 mRNA was only seen in the crypts and increased with morphological development of the crypts. In contrast Lgr5 mRNA was expressed in the crypt and the villi in the embryonic periods but became restricted to the intestinal crypt during the post-hatch period. Ki67 mRNA was expressed throughout the intestine pre-hatch, but then expression became restricted to the crypt and the center of the villi. The ontogeny of Olfm4, Lgr5, and Ki67 expressing cells show that proliferation in the peri-hatch intestine changes from along the entire villi to being restricted within the crypts.

## INTRODUCTION

The small intestine is composed of 2 major structural components. The villi, which are finger-like projections that extend into the lumen of the intestine, are responsible for nutrient absorption. The crypts, which are invaginations of intestinal tissue into the lamina propria, are populated with stem cells that produce replacement cells for the villi (Uni et al., 2000). The intestine is under constant cellular turn-over as functional epithelial cells undergo apoptosis or are sloughed off near the villi tip (Hall et al., 1994). In order to prevent loss of absorptive surface area, sufficient numbers of replacement cells must be produced by the crypt and migrate up the villi.

The small intestine at embryonic day (**e**) 15, just before the embryo consumes the amnion, is rudimentary and has minimal functional capacity to digest complex feedstuffs, as the yolk sac has been the primary nutrient absorption organ (Wong and Uni, 2021). By day of hatch, the intestine has activated and is functional enough to support consumption of exogenous feedstuff, though not efficiently (Geyra et al., 2001; Reicher et al., 2020; Reicher and Uni, 2021). By 7 d post-hatch the small intestine is morphologically mature, comparable in development to 21-day-old broilers (Van Leeuwen et al., 2004).

The late-term embryo and hatchling have cells capable of proliferation along the villi rather than restricted to the crypt as in mature intestines (Uni et al., 1998; Reicher et al., 2020, 2022). As part of the small intestinal maturation process the proliferative capabilities become restricted to the crypt (Uni et al., 2000). Recently, Reicher et al. (2020, 2022) have categorized cells as stem, progenitor, proliferative, and differentiated using immunofluorescence in chicks subjected to early, delayed feeding or in ovo feeding and have described population changes within the crypt and villi during e17 to 10 d. Other studies have looked at the in situ hybridization (**ISH**) of stem cell marker genes Olfactomedin 4 (**Olfm4**) and Leucine-rich repeat containing G protein coupled receptor 5 (**Lgr5**) in chickens during e19 to 7 d (Zhang and Wong, 2018). No study has combined quantitative gene expression and qualitative visualization to investigate proliferation in the small intestine during the peri-hatch period. Therefore, the objective of this study was to profile the changes in proliferative cells across the small intestine focusing on the development of the intestinal crypt during the last days in ovo and first 7 d post-hatch using in situ hybridization and quantitative PCR analysis of stem and proliferative cell marker genes.

## MATERIALS AND METHODS

### Animals and Tissue Collection

All procedures were reviewed and approved by the Institutional Animal Care and Use Committee at Virginia Tech. Fertile eggs (Cobb 500) were received from a local hatchery and incubated in a Natureform 1080 incubator at 37.5°C and 55% relative humidity. Chicks were pulled on d 21.5 of incubation and placed into battery cages (n = 20/cage) with ad libitum access to water and feed that met their nutritional requirements (National Research Council 1994).

On e17 and e20, 6 eggs per treatment were randomly selected for sampling. Eggs were opened and embryos were euthanized by cervical dislocation. The entire small intestine was removed intact and rinsed with cold 1% PBS. The duodenal loop (with the pancreas removed), jejunum (spanning from the end of the duodenal loop to Meckel's diverticulum), and ileum (spanning from Meckel's to the ileocecal junction) were separated and then cut into 3 pieces. The middle piece was placed into neutral buffered formalin for fixation and later histological analysis, while the other 2 pieces were diced and snap frozen in liquid nitrogen for subsequent gene expression assays. This process was repeated with post-hatch chicks on 1, 3, and 7 d.

### Gene Expression and Statistical Analysis

Frozen diced samples were homogenized with TriReagent using a tissue lyser and total RNA was extracted using Directzol RNA mini prep columns (Zymo Research, Irvine, CA). Complementary DNA was synthesized from 1 mg of total RNA using the high capacity cDNA reverse transcription kit (Thermo-Fisher Scientific, Waltham, MA). Gene expression was determined by quantitative PCR (**qPCR**) utilizing Fast SYBR Green Master Mix on an Applied Biosystems 7500 Fast Real-time PCR system (Thermo Fisher Scientific). qPCR reactions were run in duplicate. Gene primers were designed using Primer Express 3.0 (Thermo Fisher Scientific) and are listed in Table 1. Chicken ribosomal protein L4 (**RPL4**) and ribosomal protein lateral stalk subunit P1 (**RPLP1**) were used as reference genes as they were the most stable of 4 genes assessed by NormFinder (Andersen et al., 2004). The geometric mean of RPLP1 and RPL4 was subtracted from the Ct value to obtain the ΔCt value for each sample. The average ΔCt value of the e17 duodenum was used as the calibrator to calculate ΔΔCt and fold change using the 2^−ΔΔCt^ method (Schmittgen and Livak, 2008).

**Table 1:** Primer sequences for genes.

Gene name	Forward/Reverse Primers (5’ to 3’)	Amplicon Size (bp)	Accession no.
Leucine-rich repeat containing G protein-coupled receptor 5 (Lgr5)	TGGTTTGACCTTCGTTTGCA/ GGACATATACAATGGAGATCTGAAAACT	67	XM_425441.7^1^
Olfactomedin 4 (Olfm4)	TTGCCGGATACCACCTTTCC/ TTTCTGCAAGAGCGTTGTGG	72	NM_001040463.1
Marker of Proliferation mKi67 (Ki67)	CACAGGCAAAGGCTGTCAAA/ TCCGTGCAATTTTCCTTGCT	63	XM_015289038.4^1^
Ribosomal protein large subunit P1 (RPLP1)	TCTCCACGACGACGAAGTCA/ CCGCCGCCTTGATGAG	55	NM_205322.1
Ribosomal protein large subunit 4 (RPL4)	TCAAGGCGCCCATTCG/ TGCGCAGGTTGGTGTGAA	63	NM_001007479.1

1 The primer sequences were designed for regions of consensus between all published variants.

To conform to normality requirements for statistical analysis, data were logarithmically transformed. Data are presented as back transformed means and approximated standard errors (Jørgensen and Pedersen, 1998). Gene expression data were analyzed using a factorial ANOVA considering day of age, intestinal segment and the interaction of Day × Segment using JMP v15.0 (SAS Institute Cary, NC) When ANOVA results were statistically significant (*P* ≤ 0.05) mean separation was conducted with Tukey's Honestly Significant Difference Test.

### In Situ Hybridization

The intestinal segments were placed in formalin for approximately 16 h, 70% ethanol for 24 h and then stored in fresh 70% ethanol at 4°C. The fixed segments were embedded in paraffin (StageBio, Mount Jackson, VA). Formalin fixed paraffin embedded tissues of 3 individuals per timepoint were sectioned (5 µm) using a microtome and mounted on Superfrost-Plus glass slides (Electron Microscopy Sciences, Hatfield, PA). In situ hybridization was conducted using the RNAscope (Advanced Cell Diagnostics, Newark, CA) method using single plex probes for Olfm4, Lgr5, and Ki67 (Table 1) and the RNAscope 2.5 HD Assay–BROWN or RNAscope 2.5 HD Assay- RED detection kits (Advanced Cell Diagnostics). Slides were counterstained with 50% Gill's hematoxylin no. 1 (Sigma-Aldrich, St. Louis, MO), rinsed in distilled water, and placed in 0.02% ammonia water to turn the purple stain to blue. Slides were sealed with VectaMount (Vector Lab, Burlingame, CA) for the brown kit or EcoMount (Biocare Medical, LLC, Pacheco, CA) for the red kit and a glass coverslip. All ISH was conducted in sets containing each timepoint for each gene. Brightfield microscopy images were captured using a Nikon Eclipse 80i microscope with a DS-Ri1 digital camera (Nikon Instruments Inc., Melville, NY).

## RESULTS

### Gene Expression by qPCR

**Figure 1:**
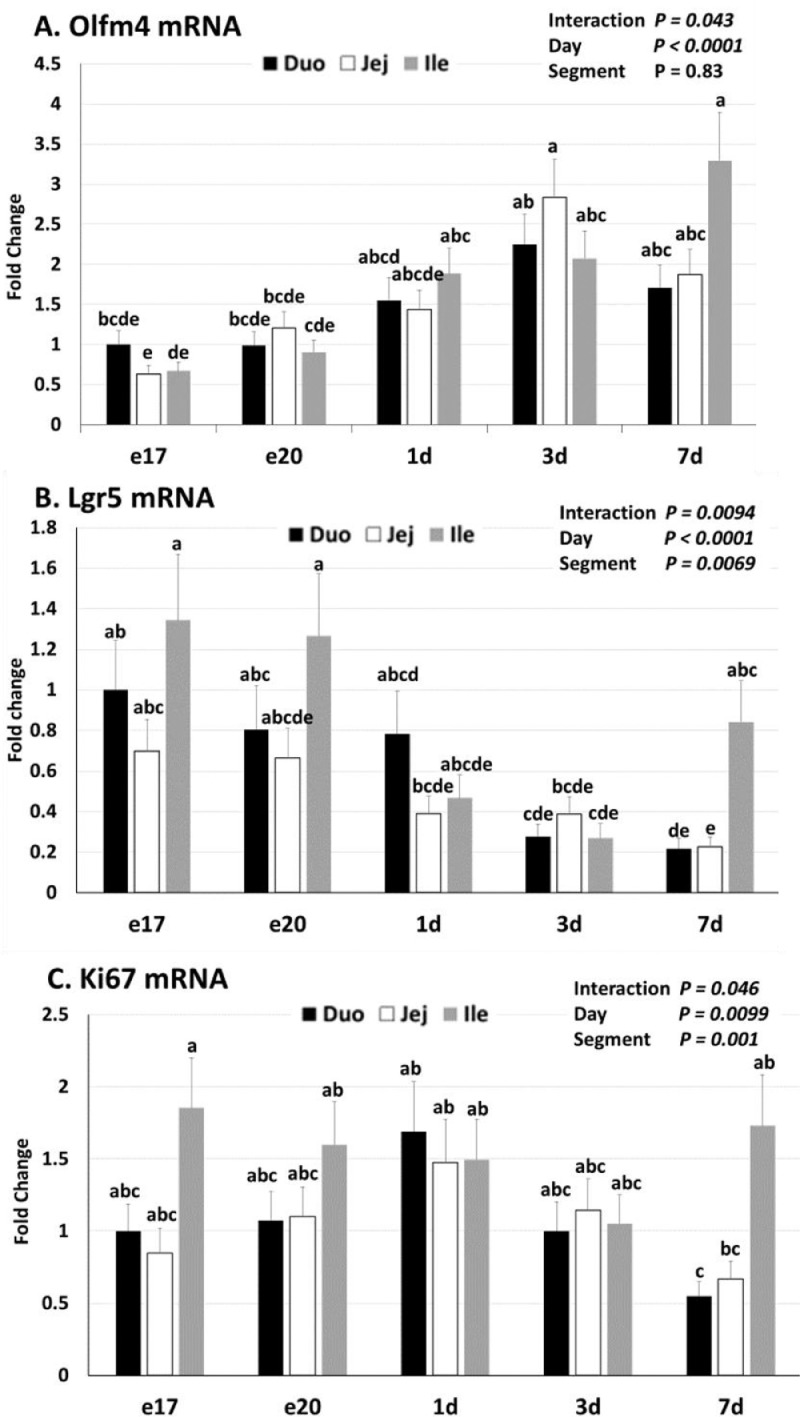
Relative mRNA abundance of Olfm4, Lgr5, and Ki67 in the small intestine during the peri-hatch period. Tissue samples were collected from duodenum (Duo), jejunum (Jej), and ileum (Ile) at embryonic days 17 (e17) and 20 (e20) and 1(1 d), 3 (3 d), and 7 (7 d) days post-hatch (n = 6). Relative mRNA abundance of olfactomedin 4 (A, Olfm4), leucine-rich repeat containing G protein-coupled receptor 5 (B, Lgr5), and marker of proliferation Ki67 (C) was determined by qPCR. The e17 duodenum was used as the calibrator for calculation of fold change. A factorial ANOVA was used to analyze for differences. Mean separation is listed as lower-case letters above bars; bars that do not share the same letters are significantly different (*P* ≤ 0.05).

The stem cell marker Olfm4 was affected by the interaction of Day × Segment (*P* = 0.043; Figure 1A). In the duodenum Olfm4 mRNA did not change with age; whereas in the jejunum Olfm4 mRNA increased from e17 to 3 d and 7 d. In the ileum Olfm4 mRNA increased from e17 to 1 d and then remained elevated from 1d to 7 d. Overall, Olfm4 mRNA increased over time (*P* < 0.0001).

The stem cell marker gene Lgr5 also varied by the interaction of Day × Segment (*P* = 0.0094; Figure 1B). In the duodenum, Lgr5 mRNA declined from e17 to 3 d and 7 d. In the jejunum, Lgr5 mRNA declined from e17 to 7 d. In the ileum, Lgr5 mRNA declined from e17 to 3 d. Overall, Lgr5 mRNA decreased over time (*P* < 0.001). In addition, there was a segment effect with Lgr5 mRNA greater in the ileum than the jejunum (*P* = 0.0069).

The proliferative marker gene Ki67 was affected by the interaction of Day × Segment (*P* = 0.046; Figure 1C). In the duodenum, Ki67 mRNA was unchanged from e17 to 1 d and then declined from 1 d to 7 d. In the jejunum and ileum, Ki67 mRNA did not change over time. Overall Ki67 mRNA followed a quadratic pattern with a peak at 1d (*P* = 0.0099).

### Gene Expression by In Situ Hybridization

**Figure 2:**
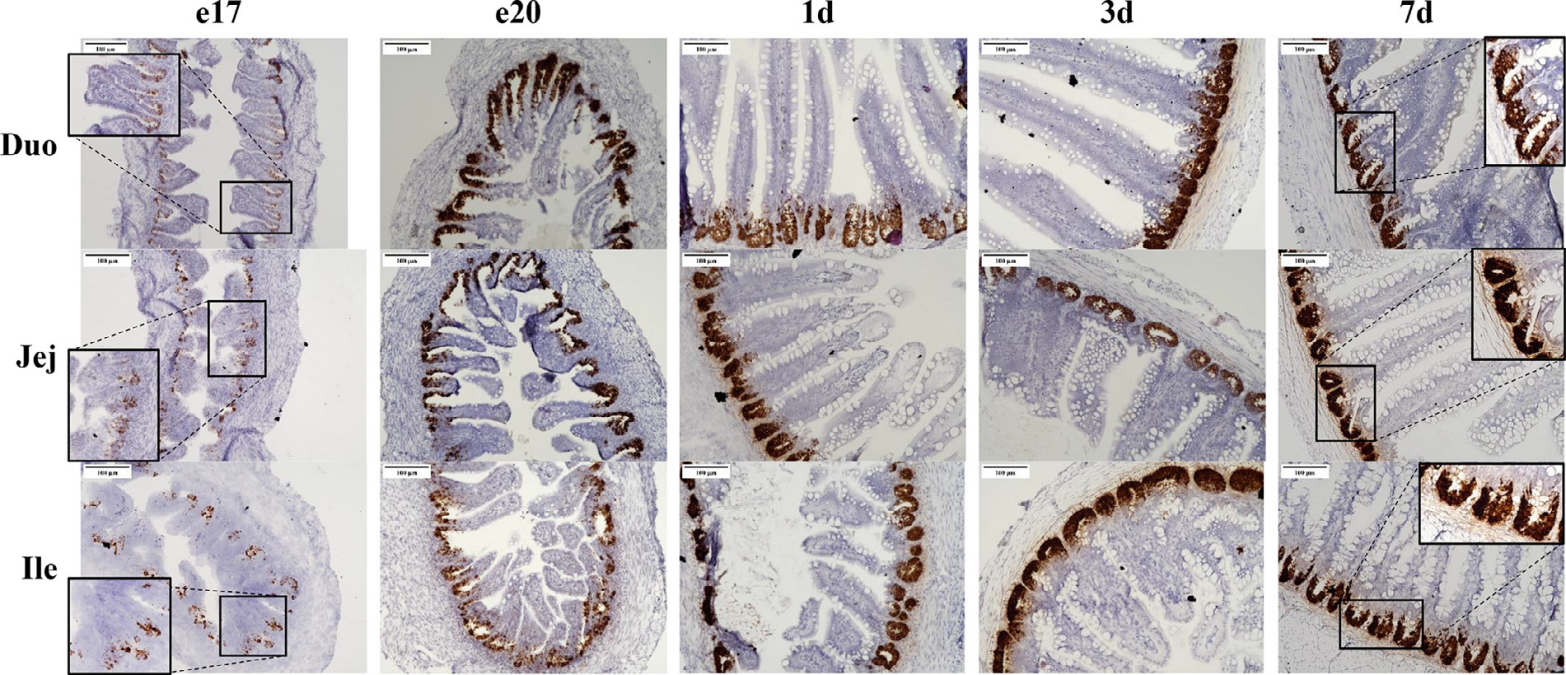
Expression of Olfm4 mRNA in the intestine from late embryogenesis to early post-hatch using in situ hybridization. Tissue samples were collected from duodenum (Duo), jejunum (Jej), and ileum (Ile) at embryonic days 17 (e17) and 20 (e20) and 1(1d), 3 (3 d), and 7 (7 d) days post-hatch (n = 3). Formalin-fixed, paraffin-embedded tissues were analyzed by in situ hybridization. Cells expressing Olfm4 mRNA (brown staining) were detected using the RNAscope 2.5 HD Assay-BROWN kit. The tissues were counterstained with 50% hematoxylin. Images were captured using 200× magnification.

The small intestine showed morphological changes from the embryonic period (e17 and e20) to the post-hatch period (1 d, 3 d, 7 d). Olfactomedin 4 mRNA was restricted to the crypts (Figure 2), and therefore is useful for highlighting crypt development. The villi developed from their initial pear-shaped structure pre-hatch to long, straight and narrow structures post-hatch resulting in an increased available surface area for nutrient absorption. The crypts had a two-stage development process. First crypts deepened into the lamina propria during the embryonic period and second during post-hatch the crypts increased in number by crypt fission, where a single crypt split into 2 or 3 crypts. This can be seen in the inset of the jejunum on 7 d (Figure 2). By 7 d the intestine was structurally the same as a mature broiler, though proportionally smaller. During the embryonic days (e17 and e20), the Olfm4 mRNA signal was less intense than at post-hatch ages, making the crypt appear speckled instead of solid brown as observed in the post-hatch samples. The Olfm4 mRNA signal had consistent appearance across all intestinal segments at a given age.

**Figure 3:**
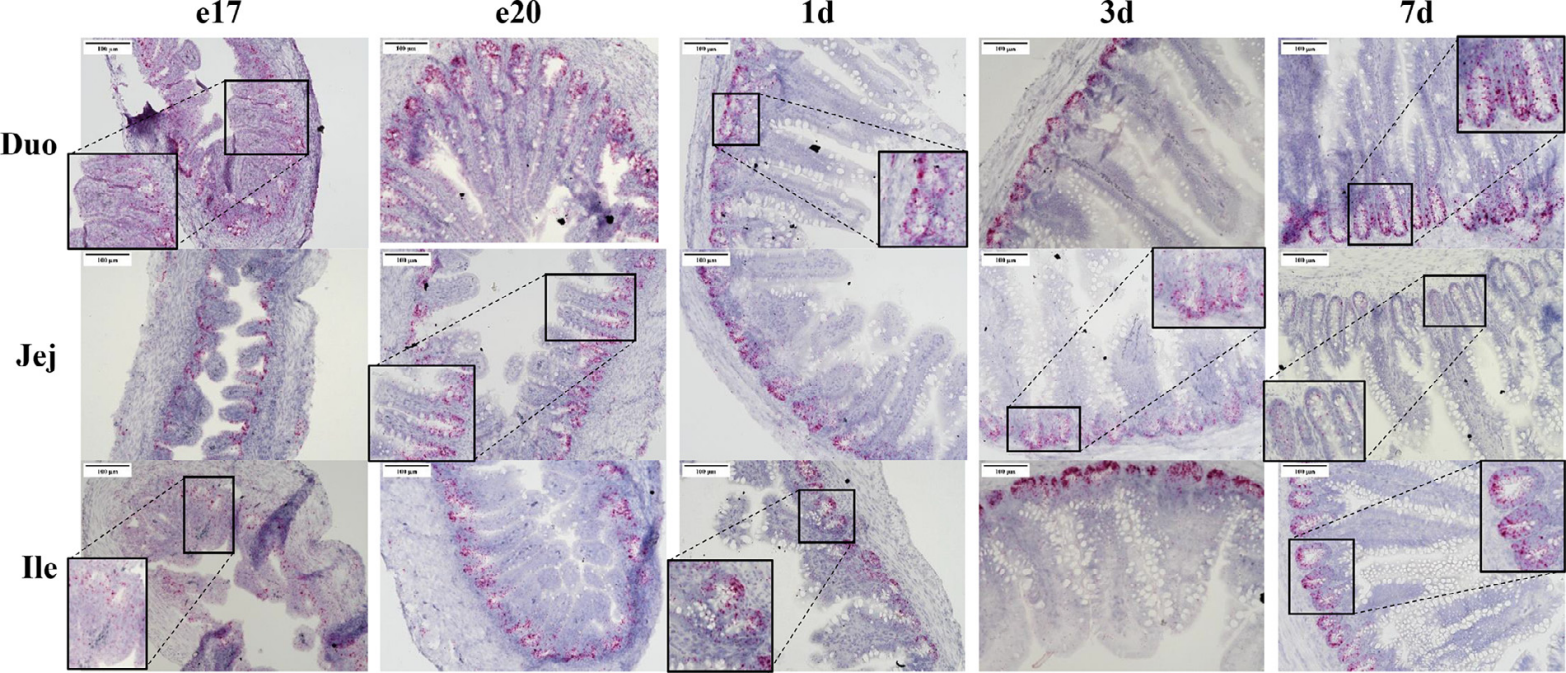
Expression of Lgr5 mRNA in the intestine from late embryogenesis to early post-hatch using in situ hybridization. Tissue samples were collected from duodenum (Duo), jejunum (Jej), and ileum (Ile) at embryonic days 17 (e17) and 20 (e20) and 1(1 d), 3 (3 d), and 7 (7 d) days post-hatch (n = 3). Formalin-fixed, paraffin-embedded tissues were analyzed by in situ hybridization. Cells expressing Lgr5 mRNA (red staining) were detected using the RNAscope 2.5 HD Assay-RED kit. The tissues were counterstained with 50% hematoxylin. Images were captured using 200× magnification.

In contrast to Olfm4, the Lgr5 signal was observed in both the crypts and the villi and appeared as single dots on the tissue rather than staining the entire region indicating reduced mRNA expression (Figure 3). Each signal dot represents a single mRNA molecule according to the manufacturer of the RNAscope procedure (Wang et al., 2012). The red chromogen was used for Lgr5 mRNA because it has a higher sensitivity than the brown chromogen. During the embryonic period, numerous areas of Lgr5 staining can be observed along the villi. However, post-hatch only a few scattered Lgr5 signals were seen outside of the crypt and only in the lower villus region. Notably the jejunum on 7 d showed extremely low expression of Lgr5 mRNA. There are only a few solitary dots visible within the crypt.

**Figure 4:**
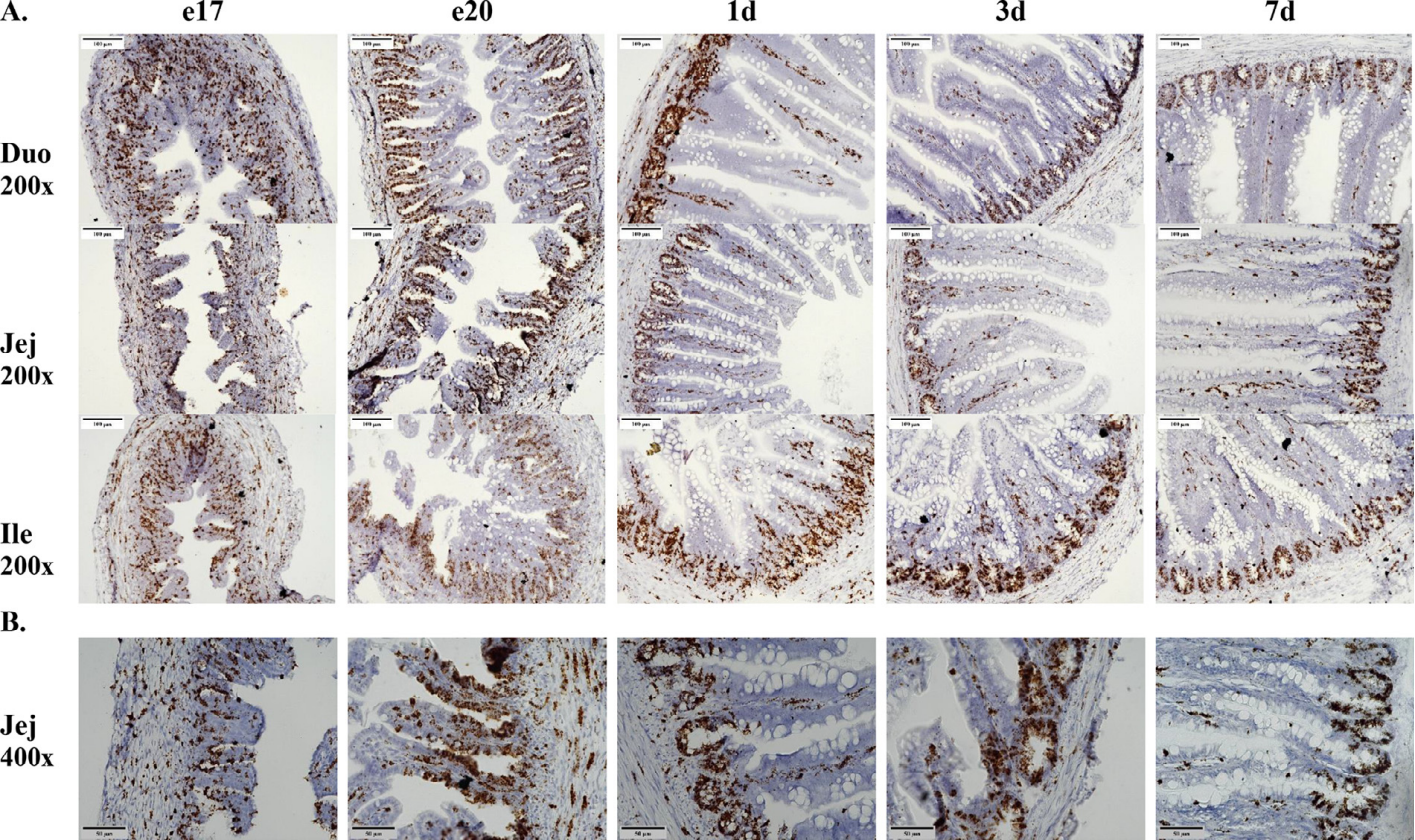
Expression of Ki67 mRNA in the intestine from late embryogenesis to early post-hatch using in situ hybridization. (A) Tissue samples were collected from duodenum (Duo), jejunum (Jej), and ileum (Ile) at embryonic days 17 (e17) and 20 (e20) and 1(1 d), 3 (3 d), and 7 (7 d) days post-hatch (n = 3). Formalin-fixed, paraffin-embedded tissues were analyzed by in situ hybridization. Cells expressing Ki67 mRNA (brown staining) were detected using the RNAscope 2.5 HD Assay-BROWN kit. The tissues were counterstained with 50% hematoxylin. Images were captured using 200× magnification. (B) The same tissue samples from the jejunum at 400× magnification.

Proliferative cells express the gene Ki67. During the embryonic period Ki67 staining appeared ubiquitous as proliferative cells were expressed in the crypt, villi, and the underlying mucosal tissue (Figure 4A). Generally, post-hatch the expression was confined to mainly the crypt and along the center of the villi. Ki67 mRNA expression was similar between intestinal segments at a given age. Not all cells in the crypt expressed Ki67 mRNA, especially during post hatch timepoints (Figure 4B). This lack of signal is especially apparent when the Ki67 staining pattern (Figure 4) is compared with the Olfm4 crypt staining (Figure 2).

## DISCUSSION

Olfactomedin 4 and Lgr5 are stem cell marker genes, which are involved in different signaling pathways that maintain the stem cell niche. Olfm4 responds to Notch signals while Lgr5 responds to Wnt signals (Demitrack and Samuelson, 2016). In mammals Lgr5 and Olfm4 are interchangeable for marking stem cells within the crypt (Koo and Clevers, 2014). Our qPCR results showed contrasting temporal patterns. Olfm4 mRNA increased post-hatch while Lgr5 mRNA decreased post-hatch, which matches the ISH results seen in this study and previously in Zhang and Wong (2018). Olfm4 is a more robust intestinal stem cell marker for chickens as it highlights the entire intestinal crypt; whereas, Lgr5 marks cells within the crypt but also some cells along the villi. The Lgr5 staining was also different from the staining with Olfm4. Olfm4 signals covered the entire cell, while Lgr5 signals were light and punctate. These differences indicate that chicken intestinal stem cells express Olfm4 mRNA to a greater extent than Lgr5 mRNA as described previously by Zhang and Wong (2018). This expression pattern differs from that of mammals (Schuijers et al., 2014).

It is unclear functionally what role Olfm4 plays within the stem cell. Olfm4 is a secreted glycoprotein that has a regulatory role for cell proliferation and apoptosis implicating the gene in innate immunity, inflammation, and cancer (Kobayashi et al., 2007; Liu and Rodgers, 2016). It is clear though that Olfm4 requires Notch signaling and that Notch signaling is required for maintaining the stem cell niche in mammals (Carulli et al., 2015; Demitrack and Samuelson, 2016). Previous research from our lab showed that Olfm4-expressing cells were absent in some cases of chickens clinically diagnosed with Runting Stunting Syndrome (Cloft et al., 2022). Interestingly in those cases, the crypt cells continued to normally express sodium glucose transporter 1, Ki67, and mucin-2 mRNA, suggesting that the crypt remained functional despite the absence of Olfm4 expression. Similarly, Schuijers et al. (2014) reported that the deletion of Olfm4 in mice caused no change in intestinal phenotype, which suggests a limited functionality of Olfm4. Thus, it seems that while Notch signaling is required for crypt development and functionality, Olfm4 expression may not be required.

In contrast, the specific role of Lgr5 within the mammalian crypt is established. Lgr5 is a receptor that responds to Wnt signals that construct and maintain the crypt stem cell niche (Sato et al., 2011). In chickens, Zhang and Wong (2018) showed that the Lgr5 signal was also present in vascular endothelial cells of the yolk sac of chick embryos, leading them to suggest that Lgr5 is a general stem cell marker gene rather than a specific intestinal stem cell marker gene. The decrease in Lgr5 mRNA as quantified by qPCR over time is likely due to the loss of Lgr5 expression outside of the crypts. This suggests that the growth of the villi during the embryonic period may be mediated by cells with stem cell-like properties. These cells then diminish post-hatch, leaving only stem cells in the crypt.

Ki67 expressing cells were observed in the crypt and along the center of the villi where in mammals the central lacteal and capillary bed resides. The observed Ki67 mRNA in the center of the villi in chickens is likely for proliferation of cells within the capillary bed underlying the epithelial layer. The mRNA abundance of Ki67 as quantified by qPCR did not change greatly over time in this study, which indicates the continuous need for proliferative cells for repair or growth of the villi.

Another marker of cell proliferation is proliferating cell nuclear antigen (**PCNA**). Previously, Geyra et al. (2001) reported that the percentage of PCNA-expressing cells in the crypt increased from 50 to 60% over the first 48 h post-hatch and during that same period decreased along the villi from 20 to 10%. This finding reiterated an earlier report of similar PCNA expression distribution by 108 h post-hatch (Uni et al., 2000).

Utilizing the proliferative marker genes PCNA and SRY-box transcription factor 9 (**Sox9**), Reicher et al. (2020, 2022) classified stem cells within the crypt as positive for Sox9 but negative for PCNA, while progenitor cells were positive for both genes, and proliferating cells were negative for Sox9 but positive for PCNA. In both studies, more crypt cells were classified as progenitor cells rather than stem cells. Along the villi PCNA was detected in the lower villi region, while Sox9 was not expressed anywhere along the villi. It is unclear exactly how Sox9, PCNA, and Ki67 overlap in the chicken, but in mammals they are described as somewhat overlapping and can be used interchangeably in intestinal tissue (Blache et al., 2004; Verdile et al., 2019).

During the first few days post-hatch the intestine rapidly increases in size (Schmidt et al., 2009), likely necessitating widespread proliferative capacity. Based on Ki67 mRNA signals, in agreement with studies by Reicher et al. (2020, 2022), it seems that cells along the villi are proliferating during the late embryonic period enabling rapid growth. After hatch, the expression of Ki67 mRNA was mainly localized to the crypts, consistent with previous work by Geyra et al. (2001) and Uni et al. (2000). The restriction of proliferation in the crypt coincides with increased nutritional capabilities of the chick and an increase in mature enterocytes (Moran, 2007; Reicher and Uni, 2021).

In conclusion, this study profiles the stem cell marker genes Olfm4 and Lgr5 along with the proliferative cell marker gene Ki67 in the small intestine of peri-hatch broiler chickens. During the peri-hatch period the intestine transitions from primarily proliferative to mature intestinal functions. Olfm4 mRNA increased post-hatch while Lgr5 mRNA decreased post-hatch using both qPCR and ISH. Olfm4-expressing cells were only seen in the crypts. Lgr5-expressing cells changed from expression along the villi, in the crypt and in the lamina propria during late embryogenesis to expression restricted to the intestinal crypt post-hatch. Ki67-expressing cells appeared throughout the intestine during the pre-hatch period, but became restricted to the crypts and along the center of the villi post-hatch. The restriction of proliferative cells to the crypts is likely essential for the successful transition to post-hatch life as functional absorptive and secretory cells are differentiated from stem cells exiting the crypt.
